# Observed metabolic asymmetry within soybean root nodules reflects unexpected complexity in rhizobacteria-legume metabolite exchange

**DOI:** 10.1038/s41396-018-0188-8

**Published:** 2018-06-13

**Authors:** Dušan Veličković, Beverly J. Agtuca, Sylwia A. Stopka, Akos Vertes, David W. Koppenaal, Ljiljana Paša-Tolić, Gary Stacey, Christopher R. Anderton

**Affiliations:** 1 Environmental Molecular Sciences Laboratory, Earth and Biological Sciences Directorate, Pacific Northwest National Laboratory, 902 Battelle Boulevard, Richland, WA 99354 USA; 2 Divisions of Plant Sciences and Biochemistry, C. S. Bond Life Sciences Center, University of Missouri, Columbia, MO 65211 USA; 3 Department of Chemistry, The George Washington University, Washington, DC 20052 USA

## Abstract

In this study, the three-dimensional spatial distributions of a number of metabolites involved in regulating symbiosis and biological nitrogen fixation (BNF) within soybean root nodules were revealed using matrix-assisted laser desorption/ionization mass spectrometry imaging (MALDI-MSI). While many metabolites exhibited distinct spatial compartmentalization, some metabolites were asymmetrically distributed throughout the nodule (e.g., S-adenosylmethionine). These results establish a more complex metabolic view of plant–bacteria symbiosis (and BNF) within soybean nodules than previously hypothesized. Collectively these findings suggest that spatial perspectives in metabolic regulation should be considered to unravel the overall complexity of interacting organisms, like those relating to associations of nitrogen-fixing bacteria with host plants.

The symbiotic association between nitrogen-fixing soil bacteria (Rhizobiaceae) and plants of the family Leguminosae generate specialized organs called root nodules [1]. Elucidating metabolic processes within these plant organs, where biological nitrogen fixation (BNF) occurs, is essential for developing more sustainable agricultural practices, for example. Generally, there are two classes of nodules: (i) indeterminate nodules, such as those formed on alfalfa or pea, and (ii) determinate nodules, such as those formed on soybean or Lotus. Indeterminate nodules retain a terminal, apical meristem, and have been extensively studied, including by MALDI-MSI [2, 3]. This is in part because the full ontogeny of nodule development—from apical infection, bacteroid differentiation, nitrogen fixation, and basal senescence—is preserved longitudinally [4]. In contrast, determinate nodules lack an apical meristem and develop in principle by cellular expansion after invading rhizobia induce initial plant cell division. The result is that the globular soybean nodule does not preserve the preceding infection events. Accordingly, this has led in large part to the simplified view that soybean nodules are basically uniform in their metabolism, albeit with the presence of microscopically distinct compartments—i.e., outer cortex, inner cortex, and infection zone [4]. Recently, our group profiled the metabolome of intact soybean root nodules along with its individual biological components using laser ablation electrospray ionization mass spectrometry [5]. This method, nevertheless, provided limited spatially resolved metabolic information on the anatomical compartments of the nodule. On the contrary, the high (spatial and mass)-resolution molecular tomography described in the present study revealed unexpected complexity in the soybean nodule metabolism.

Herein, we spatially resolved the distribution of an array of metabolites within soybean nodules, using matrix-assisted laser desorption/ionization Fourier transform ion cyclotron resonance mass spectrometry imaging (MALDI-FTICR-MSI) [6]. The value of this methodology is illustrated in Supporting Fig. 1. Among the approximately 140 annotated metabolites, most were co-localized within distinct anatomical compartments (Supporting Figs. 2–3, Supporting Tables 1–3). However, a few of the metabolites, including S-adenosylmethionine (SAM) and ADP (Fig. 1), showed a pronounced asymmetric distribution throughout the central zone of the nodule. This finding contradicts the long-standing hypothesis about metabolic homogeneity of this region within the soybean nodule [4], and points to a previously unacknowledged biochemical complexity in symbiotic plant–microbe interactions. For example, *heme* B, an essential molecule for providing microaerobic conditions during BNF [7], maintains a symmetric distribution within the infection zone, radially decreasing in abundance (Fig. 1b, bottom). Whereas the asymmetric distribution of SAM throughout the infection zone sheds a different light in its role in downstream BNF processes, given that SAM occupies a central role in both polyamine [8] and phosphatidylcholine (PCs) biosynthesis [9], molecules involved in nodule growth [8] and rhizobia-plant recognition [9], respectively. Further, ADP plays a central role during BNF, and its abundance is a key indicator of the energetic and oxidative state within the nodule [10]. Interestingly, it seems that ADP and SAM share the same distribution pattern (Pearson correlation coefficient of 0.80, Supporting Table 4), which is perhaps a consequence of ADP’s involvement in SAM biosynthesis [11]. We additionally tested the importance of SAM and ADP during BNF by imaging the relative abundance and distribution of these metabolites in nodules inoculated with mutant rhizobia that could not efficiently fix nitrogen (*nif*H^−^) (Fig. 1c). As expected, *heme* B presence in *nif*H^*−*^ nodules was decreased compared to wild-type (WT) strain, given that the observable reddish pigment indicative of leghemoglobin presence is significantly diminished in the *nif*H^*−*^ infection zone in comparison to the infection zone of the WT strain (Supporting Fig. S4). Additionally, compared to WT, the *nif*H^−^ mutant produces substantially less SAM, and the noticeable divergence in ADP content and distribution between *nif*H^−^ and WT nodules confirms their different energetic requirements. Other metabolites that show noticeable divergence between WT and *nif*H^−^ mutant soybean nodules can be found in Supporting Fig. S5.

**Fig. 1:**
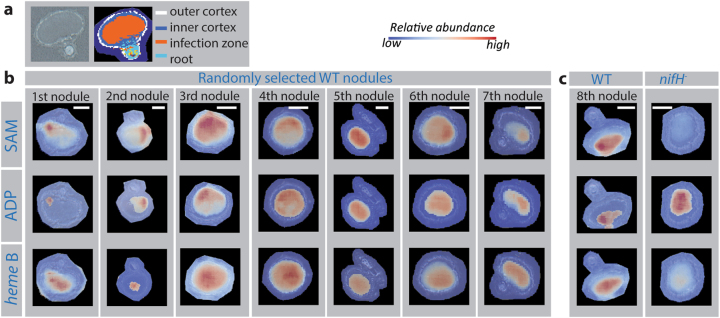
**a** Anatomy of the soybean nodule as viewed through an optical image of a section (left) and the MALDI spectral spatial segmentation, which distinguishes areas based on their spectral composition (right). **b** Distribution of SAM, ADP, and *heme* B in the central cross-section of seven randomly selected WT nodules. Nodules were analyzed in different experiments to minimize analytical bias. In nodule No 5, SAM and *heme* B show symmetrical distribution pattern, which suggest that asymmetry in nodule metabolism can be consequence of nodule development over time. **c** For SAM, ADP, and *heme B* imaged in WT and *nif*H^−^ nodules, both nodules were imaged in the same experiment, so that the relative intensity of ion signals can be compared. Scale bars are 1 mm

Further exploration of metabolic distributions within soybean root nodules was performed by molecular tomography (Fig. 2e, f). Here, conventional two-dimensional (2D) MALDI-FTICR-MSI analyses of serial sections traversing through the entire soybean nodule were acquired and subsequently these images were reconstructed into a three-dimensional (3D) MALDI-FTICR-MS image (Fig. 2a) [12]. Tomographically, three distinct microscopic compartments were readily visualized by mapping characteristic metabolites (Fig. 2b). Further, tomography revealed that SAM asymmetry (Fig. 2c) was consistent throughout the organ, whereas *heme* B is symmetrically localized throughout the infection region (Fig. 2d).

**Fig. 2:**
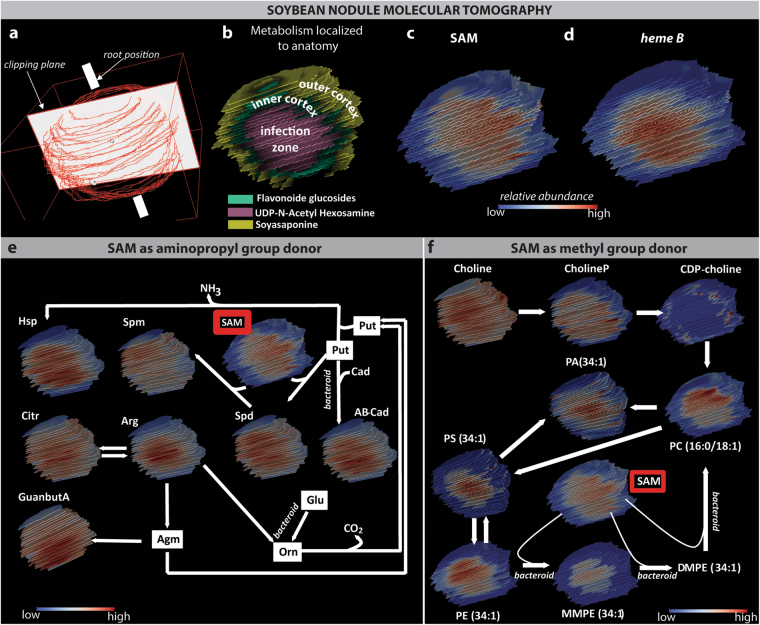
3D-MALDI-FTICR-MSI of soybean root nodule metabolism. **a** Scheme illustrating the construction of the tomography image from 2D images. Resulting 3D localization of (**b**) three microscopic anatomical regions imaged by characteristic compounds, where UDP-N-acetyl hexosamine is co-localized with the infection zone, flavonoid glycoside is co-localized with the inner cortex, and soyasaponin is located within the outer cortex of the soybean nodule. The 3D distribution of (**c**) SAM and **d** *heme* B within the soybean root nodule. Mapping the 3D distribution within soybean nodules of the two metabolic pathways involving SAM during BNF: **e** Polyamine biosynthesis and (**f**) Phosphatidylcholine biosynthesis. For (**f**), we mapped the PC (34:1) as an example because we observed the highest number of phospholipid classes with this fatty acid composition. For both (**e**, **f**), pathway steps known to occur only in bacterium are annotated

To elucidate possible causes for the asymmetric distribution of SAM, molecules within the two main metabolic pathways where SAM is involved during BNF were examined (Fig. 2e, f). Here, the distribution of molecules involved in polyamine biosynthesis exhibit an approximately uniform or centralized pattern throughout the nodule infection zone (Fig. 2e). Notably though, spermine that is a direct product of aminopropylation transfer via SAM has an asymmetric distribution through the nodule volume, which is a pattern not observed in the 2D images. As such, sectioning was presumably performed in-plane of the uniform concentration of this molecule with respect to the nodule anatomy, which further highlights the importance of molecular tomography for even ostensibly symmetrical systems. To visualize the involvement of SAM in the fate and pathway of PC biosynthesis (Fig. 2f), we used the example of PC (34:1). There are two metabolic routes to the synthesis of PCs in legume nodules [9]: the CDP choline route, a known pathway in plant cells, and the successive methylation route, which is the only known pathway of PC synthesis in rhizobacteria. Our results suggest that PC synthesis through CDP-choline is more prominent in the cortex tissue. Thus, PC from the infection zone seems to arise mainly from bacteroid metabolism through a successive methylation of PE, where SAM serves as a methyl donor. Nonetheless, there is some divergence in SAM and PC localization that might be the result of PC turnover and interconversion into other PLs, and/or a function of the numerous pathways where SAM is utilized as a methyl group donor. Beside PCs, we observed asymmetry in phosphatidylethanolamine (PE) distribution. However, spatial asymmetry is lost as PE (34:1) is converted into phosphatidylserine (PS), and as the lipid head groups are removed from PS, PE, and PC during breakdown to phosphatidic acid (PA). This spatial complexity suggests that the membrane composition of bradyrhizobial species and surrounding plant cells is well controlled within the soybean nodule system, and that SAM might have a crucial role in this regulation.

In summary, our results demonstrate the utility of high-resolution spatial metabolomics methods, like molecular tomography via MSI, for elucidating the overall complexity of interacting organisms. While molecular tomography has previously been utilized to map metabolic distributions in single-organism systems (e.g., mouse brain [13] or mouse lung [14]), our results demonstrate that this methodology holds particular promise for the study of plant–microbe processes. Especially, where non-imaging modalities can conceal or hint at distributional differences, or when tracking metabolic routes throughout a plant system is desired. Our specific application of this imaging modality to soybean nodules uncovered previously unknown spatial complexity in nodule metabolism, which clearly plays an important role in the ability of these structures to contribute to soybean nitrogen use, and therefore crop productivity and sustainability.

## Electronic supplementary material

Supplementary Information
