# Supplementary material for: Observed metabolic asymmetry within soybean root nodules reflects unexpected complexity in rhizobacteria-legume metabolite exchange
Source: ISME J. 2018 Jun 13;12(9):2335–8. doi: 10.1038/s41396-018-0188-8 (PMC6092352; doi:10.1038/s41396-018-0188-8)
Supplement: Supplementary file 1 — Supplementary Information [file 41396_2018_188_MOESM1_ESM.docx]

**Supplemental material for**

**Observed metabolic asymmetry within soybean root nodules reflects unexpected complexity in rhizobacteria-legume metabolite exchange**

Dušan Veličković^1^, Beverly J. Agtuca^2^, Sylwia A. Stopka^3^, Akos Vertes^3^, David W. Koppenaal^1^, Ljiljana Paša-Tolić^1^, Gary Stacey^2^, Christopher R. Anderton^1^

^1^ Environmental Molecular Sciences Laboratory, Earth and Biological Sciences Directorate, Pacific Northwest National Laboratory, 902 Battelle Boulevard, Richland, WA USA 99354;

^2^ Divisions of Plant Sciences and Biochemistry, C. S. Bond Life Sciences Center, University of Missouri, Columbia, MO USA 65211;

^3^ Department of Chemistry, W. M. Keck Institute for Proteomics Technology and Applications, The George Washington University, Washington, DC USA 20052

[*Christopher.Anderton@pnnl.gov](mailto:*Christopher.Anderton@pnnl.gov); 902 Battelle Boulevard, Richland, WA USA 99354; 509-371-7970

**Text Summary**

**Experimental procedures:** Plant growth, Sample preparation for MALDI-MSI, MALDI-MSI, and Metabolite identification

**Figure S1.** Demonstrating the utility of high mass resolution and mass accuracy measurements provided by MALDI-FTICR-MSI for differentiating metabolites in situ.

**Figure S2.** Distribution of identified small molecules and secondary metabolites through the central section of soybean nodule, as revealed with MALDI-FTICR-MSI.

**Figure S3.** Distribution of identified phospholipids through the central section of the soybean nodule, as revealed with MALDI-FTICR-MSI.

**Figure S4.** Optical images of cross-sections of WT and *nif*H- soybean nodules.

**Figure S5.** Distributions and abundances of metabolites that are measurably different between WT and *nif*H^-^ mutant soybean nodules.

**Table S1.** Peak assignments in positive-ion mode profiling MALDI-FTICR mass spectra of soybean root nodules.

**Table S2.** Peak assignments in negative-ion mode profiling MALDI-FTICR mass spectra of soybean root nodules.

**Table S3.** MALDI-FTICR-MSI metabolic coverage of some pathways in soybean root nodule based on SoyKB database.

**Table S4.** The average Pearson’s correlation coefficients of SAM, ADP, and *heme* B.

**EXPERIMENTAL PROCEDURES**

**Plant Growth**

Rhizobial cells (*Bradyrhizobium japonicum*) USDA110 wild-type (WT) and fix-mutant H1 (*nif*H-) were inoculated into HM medium ([Cole and Elkan, 1973](#_ENREF_3)) (HEPES, 1.3 g/L; MES, 1.1 g/L; Na_2_HPO_4_, 0.125 g/L; Na_2_SO_4_, 0.25 g/L; NH_4_Cl, 0.32 g/ L; MgSO_4_•7H_2_O, 0.18 g/L; FeCl_3_, 0.004 g/L; CaCl_2_•2H_2_O, 0.013 g/L; yeast extract, 0.25 g/L; D-Ara, 1 g/L; sodium gluconate, 1 g/L; and pH 6.6), supplemented with 25 mg/L of tetracycline and 100 mg/L of spectinomycin for wild-type and 100 mg/L of kanamycin and spectinomycin for *nif*H*-*. The cells were then incubated and maintained for 2 d at 30 °C in an orbital shaker (MaxQ400, Thermo Scientific, Waltham, MA) set to 180 rpm. Once cellular growth reached 10^8^ cells/mL, as measured by optical density (OD600= 0.8), the culture was centrifuged at 800 × g for 10 min, washed three times with DI water, and used for seedling inoculation. Soybean seeds (*Glycine max* Williams 82), sterilized with 20% (v/v) bleach for 10 min and rinsed five times in sterile water, were planted into pots containing a mixture of autoclaved 3/1 vermiculite/perlite, respectively. The plants were grown in a greenhouse at 30 °C with a 16 h light/8 h dark cycle, and at day 3 the seedlings were inoculated with 1 mL of *B. japonicum* suspension per seedling on soil. At day 21 of growth, the roots with attached nodules were freshly harvested, plunged into liquid nitrogen, and stored at -80 °C until further use.

**Sample preparation for 2D and 3D MALDI-MSI**

Small root sections with attached nodule and individual nodules (without attached root) were excised from frozen soybean roots with a razor blade. These were individually embedded in 2.5 % carboxymethyl cellulose (CMC) and quickly frozen on a bed of dry ice. A carbohydrate rod (spaghetti) was embedded next to individual nodules to serve as positional marker during 3D image reconstruction. Embedded tissue was then mounted and cryosectioned (CryoStar NX70, Thermo Scientific), where the sample chuck and cutting blade were maintained at -13 °C and -16 °C, respectively. 10 µm thick tissue sections were taken orthogonal to length of the root or carbohydrate rod. The sections were thaw-mounted onto indium tin oxide (ITO) glass slides (BrukerDaltonics). For comparing the metabolite distribution pattern between different WT nodules, the central cross-sections of seven different nodules from different growth batches were mounted on ITO slides, so that sections from only one sample type are present on one ITO slide. For comparison of nifH- and WT strains, two random batches of both nifH- and WT strains were compared by randomly picking one nodule from each batch (four nodules in total). The central cross-sections of each nodule pair (WT and nifH-) were mounted on the same ITO slide and analyzed in the same imaging run. This was repeated for the for the second pair of samples. For 3D MSI, one of every fifth section from the top to bottom of the nodule was mounted, where a total of ~30 sections per ITO slide were mounted. Each section was mounted facing towards the side previously in contact with rest of the tissue, as to avoid positioning error of 180° during stacking of 2D images.

Application of MALDI matrix was performed using HTX TM-Sprayer (HTX Technologies, Chapel Hill, NC, USA) ([Gemperline et al., 2015](#_ENREF_4), [Anderton et al., 2016](#_ENREF_1)). DHB (2, 5-dihydroxybenzoic acid) and norharmane were used for positive and negative ion analysis mode, respectively. For DHB, 40 mg/mL in 50% MeOH was sprayed with 16 passes at 50 µL/min at 80 °C with spray spacing of 3 mm. For norharmane, 7 mg/mL in CHCl_3_:MeOH (2:1) was used, and seven passes were sprayed at 120 µL/min and 30 °C, with a spray spacing of 2 mm. A spray pressure of 10 psi (N_2_), a spray velocity of 1200 mm/min, and a sprayer nozzle distance from the sample of 40 mm was maintained for all samples.

**MALDI-MSI**

Mass spectrometry imaging was performed on a 15T MALDI-FTICR-MS (Bruker Daltonics) equipped with a SmartBeam II laser source (355 nm, 2 kHz). Data were collected in four different modes: optimized for m/z 92-500 (for low m/z values) and optimized for m/z 400-2000 (for high m/z values) in both positive and negative polarity. External calibration of instrument was performed using TuneMix (Agilent), resulting in mass measurement accuracy typically within 1 ppm across the entire m/z range. The laser was stepped across the sample in 50 µm increments (accumulating 200 laser shots per step), and because images were acquired from every fifth section, our lateral resolution for all measurement was 50 µm in all three dimensions. Image data was acquired using FlexImaging (v 4.1, Bruker Daltonics). Compass DataAnalysis was used for recalibration of acquired spectra using MALDI matrix peaks as internal calibrants. Deisotoping of mass spectra was performed using mMass 5.5.0 software. Additional image processing (i.e., peak alignment, segmentation, determining co-localized m/z values, and calculation of Pearson correlation coefficients) and visualization of image data were performed using SCiLS Lab (GmbH, Bremen, Germany). 3D MALDI images were created using the additional 3D tool feature in SCiLS Lab. All images were normalized to the total ion current.

**Metabolite identification**

Metabolites were identified by matching accurate mass (mass accuracy < 1 ppm) with the METLIN database (Supplementary Table 1 and 2), relying both on available literature coverage of legume nodulation metabolites ([Brechenmacher et al., 2010](#_ENREF_2), [Vauclare et al., 2013](#_ENREF_12), [Ye et al., 2013](#_ENREF_13), [Gemperline et al., 2015](#_ENREF_4)), and LAESI-ion mobility separation-MS and tandem MS analysis of soybean nodules performed by our group previously ([Stopka et al., 2017](#_ENREF_9)). Additional confirmation of molecular formulas was based on correlating the ion images of the monoisotopic peak with that of the naturally abundant isotopic peaks of the same molecule ([Palmer et al., 2017](#_ENREF_7)).


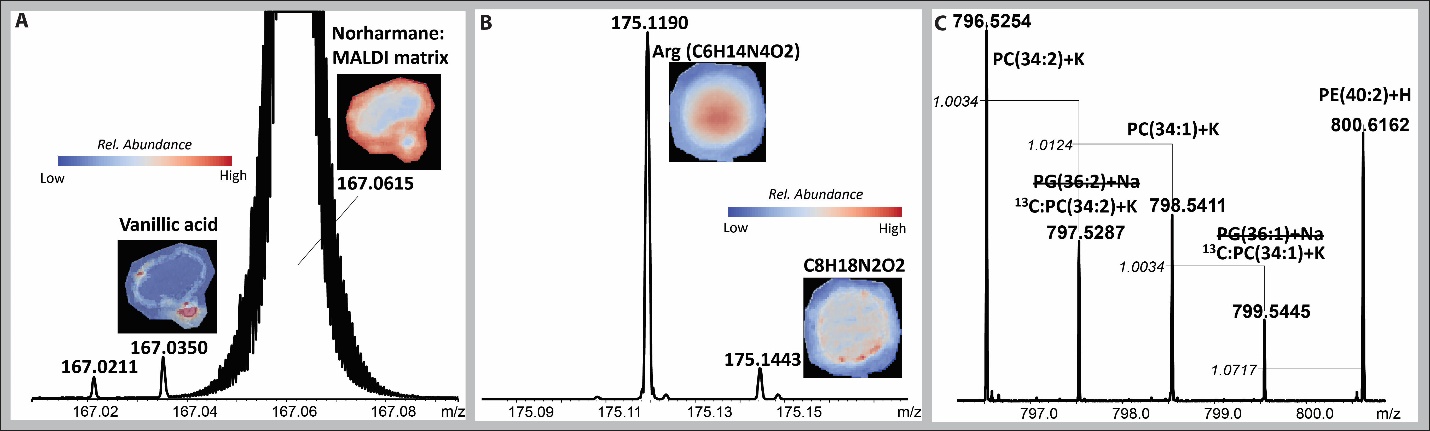


**Figure S1.** Demonstrating the utility of high mass resolution and mass accuracy measurements provided by MALDI-FTICR-MSI for differentiating metabolites in situ, where (**a**) vallinic acid was resolvable from the matrix peak of norharmane (matrix interference with analytes of interest is common with lower resolving power mass analyzers), (**b**) resolving metabolites with same nominal mass but different spatial distributions is easier, and (**c**) matching detected peaks with naturally occurring isotopes and their relative abundances can provide higher confidence in molecule annotations.

Recent introduction of ultrahigh performance mass spectrometers within imaging workflows has provided a significant advance in MSI, by allowing imaging of small metabolites more attainable with the high mass resolving power and mass accuracy these mass analyzers provide. In our work, the high mass accuracy and resolution of the 15T FTICR-MALDI-MSI enabled us to resolve metabolites with the same nominal masses, and thus to unambiguously identify elemental composition (molecular formula) of any signal of interest. The benefit of ultrahigh resolution and mass accuracy used in our approach (Supplemental Figure 1) is demonstrated through three important imaging issues which impede analysis using lower resolving power instruments ([Gemperline et al., 2015](#_ENREF_4)). First, we were able to analyze in the low m/z range (Supplemental Figure 1a), where highly abundant MALDI matrix-related signals are resolvable from some endogenous signals. One of the examples is imaging of vanillic acid, m/z 167.0351 [M-H]. With a lower resolution MS analyzer, this signal would be convoluted with the dominant signal of norharmane, which we used as the MALDI matrix in negative ion mode, and thus it would be missed. Second, compounds with the same nominal masses can more readily be resolved. An example is arginine (m/z 175.1190, [M+H]) and an unknown signal about 25 mDa apart (m/z 175.1443), which could be ascribed a molecular formula of C_8_H_18_N_2_O_2_ [M+H]. There is noticeable difference in the localization of these two metabolites, where arginine is present with highest abundance in central part of the nodule, while the signal at m/z 175.1443 co-localizes with the nodule cortex. Without ultrahigh mass resolution, those two metabolites would be present under one m/z signal and, perhaps, wrongly attributed solely to arginine (Supplemental Figure 1b). Third, ultrahigh mass accuracy enables us to discriminate between isotopic forms and the degree of saturation of phospholipids, Supplemental Figure 1c. An example is an ion at m/z 797.5287, which could be assigned to the sodium adduct of phosphatidylglycerol PG (36:2) (theoretic m/z 797.5303, hence a mass error of 2.01 ppm between measured and theoretical values). However, high accuracy analysis reveals that this ion is actually the ^13^C isotopic form of phosphatidylcholine PC (34:2) [M+K], which is present as a monoisotopic ion at m/z 796.5254. The mass difference of 1.0034 Da is exactly the same as the difference between ^13^C and ^12^C isotope, and the intensity ratio of these two peaks matches well with the simulated isotopic distribution for PC (34:2). A very similar conclusion could be derived for the ion at m/z 799.5445, which could be wrongly interpreted as PG (36:1), as opposed to the naturally abundant ^13^C­_2_- PC (34:2) [M+K] ion. Thus, it is clear that without high performance mass analyzers, imaging and identification of small metabolites and phospholipids would be very ambiguous ([Zabrouskov et al., 2001](#_ENREF_14)).

**
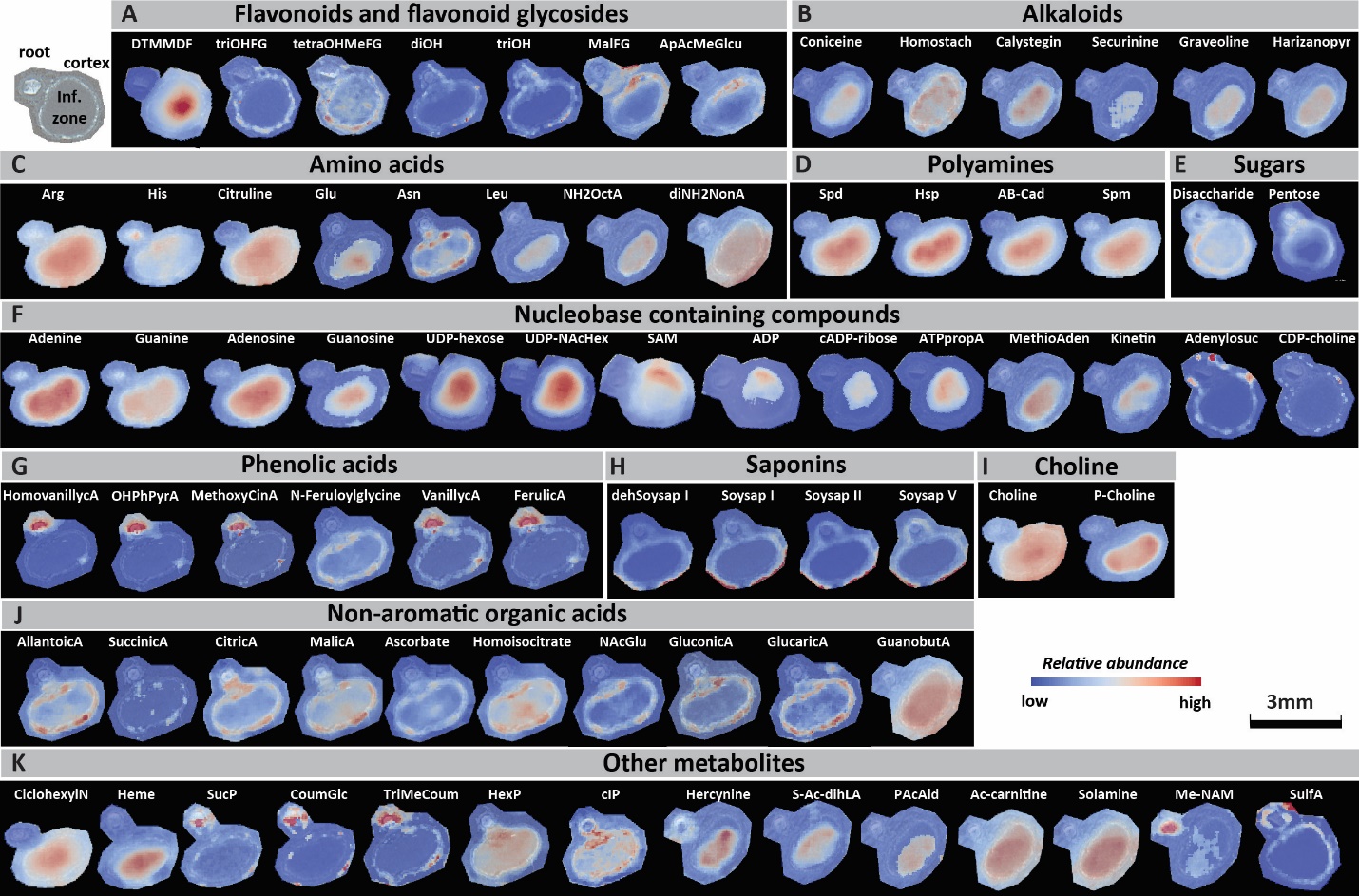
**

**Figure S2.** Distribution of identified small molecules and secondary metabolites through the central section of soybean nodules as revealed with MALDI-FTICR-MS imaging. The distribution of (**a**) flavonoids and flavonoid glycosides— *DTMMDF: dihydroxytetramethoxy methylendioxy flavone;* *triOHFG: trihydroxyflavon glucoside;* *tetraOHMeFG: tetrahydroxymethoxyflavone glucoside;* *diOHF: dihydroxyflavone;* *triOHF: trihydroxyflavone;* *MalFG: flavon malonyl glucoside; ApAcMeGlcu:Apigenin (acetyl)-methylglucuronide—, (***b**) Alkaloids, (**c**) Amino acids— *NH2OctA: aminooctanoic acid;* *diNH2NonA: diaminononanoate—*, (**d**) **b**iological polyamines—*Spd: spermidine;* *Hsp: homospermidine;* *AB-Cad: aminobutyl cadaverin;* *Spm: spermine—*, (**e**) Sugars, (**f**) nucleobase containing compounds— *SAM: S-adenosyl methionine;* *MethioAden: deoxy-(methylthio) adenosine—*, (**g**) phenolic acids— *OHPhPyrA: hydroxyohenylpyruvic acid—,* (**h**) saponins, (**i**) cholines, (**j**) non-aromatic organic acids— *NAcGlu: N-acetyl- glutamic acid—*, and (**k**) others molecules involved in diverse metabolic pathways in soybean nodule— *CyclohexylN: cyclohexylamine;* *SucP: sucrose-phosphate; CoumGlc: coumarin glucoside; triMeCoum: trimethoxycoumarines; cIP: inositol cyclic phosphate; S-Ac-dihLA: S-acetyldihydrolipoamide; PAcAldehyde:Phosphonoacetaldehyde; Ac-carnitine: acetycarnitine; Me-NAM: methylnicotineamide; SulfA: sulfuric acid—* are visualized. All images are normalized using the total ion count and color bar is adjusted for each image individually to provide best visualization of ion intensity differences. Lateral resolution is 50 µm*.* Non-standard abbreviations were explained.

To provide insights into metabolite origin and flow throughout the nodule, we imaged soybean root nodules with the root portion still attached. We found several distinct metabolite distribution patterns that could be visualized across the soybean root and adjoining nodule tissue. Specifically, a large number of metabolites show co-localization within the nodule cortex (e.g., soyasaponins, gluconic acid, CDP-choline, hydroxyphenylpyruvic acid), a few metabolites were more abundant in the root portion than in the nodule itself (e.g, phenolic acids, trimethoxycoumarin, methylnictonineamide), and some of metabolites show similar abundance in both root vascular and nodule tissues (e.g., histidine, guanine, hexoso-phosphate). Moreover, there are a number of metabolites that were not observed in the root portion. These include several metabolites that are highly concentrated in the center of the nodule (e.g., dihydroxytetramethoxy methylendioxy flavone [DTMDF], UDP-hexose, UDP-N-Acetyl-hexosamine, *heme* B), and are uniformly spread throughout the whole infection zone (e.g., arginine, adenine, choline, guanidinobutyric acid).

**
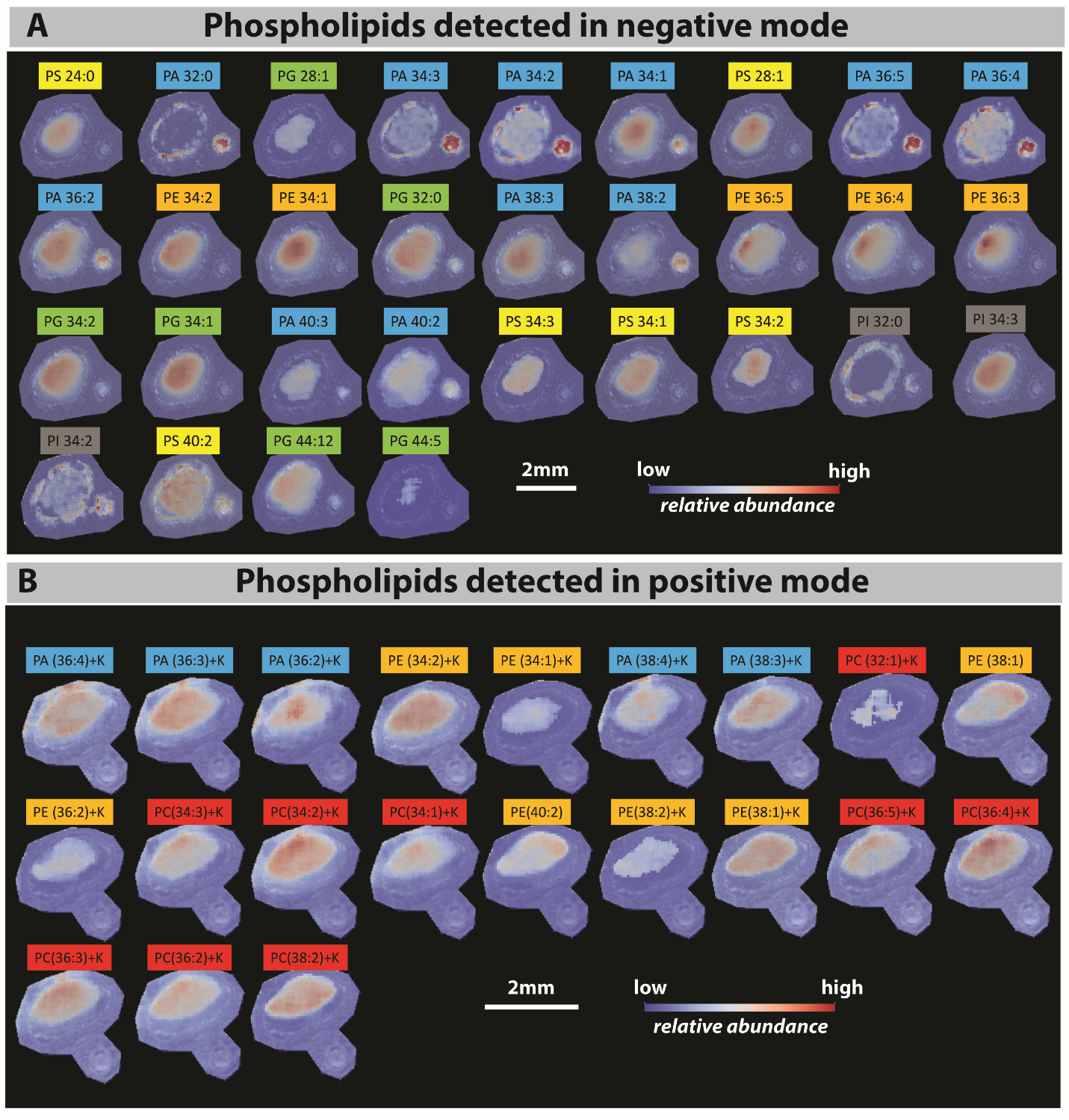
**

**Figure S3.** Distribution of identified phospholipids through the central section of the soybean nodule. (**a**) the phospholipids detected in negative mode as [M-H]^-^ ions and the (**b**) phospholipids detected in positive mode as [M+K]^+^ or [M+H]^+^ ions are shown. All ion images are normalized using the total ion count (TIC) and color bar is adjusted for each ion image individually to provide best visualization of ion intensity differences. Lateral resolution is 50 µm. Each phospholipid class is highlighted with a unique color: yellow: phosphatidylserines (PS); blue: phosphatidic acids (PA); green: phosphatidylglycerols (PG); orange: phosphatidylethanolamines (PE); red: phosphatidylcholines (PC); gray: phosphatidylinositol (PI).

The majority of species detected in both positive and negative mode MALDI-MS (grouped between 700 and 900 *m/z*) originate from phospholipids (PL). These molecules are important constitutes of cell membranes and have been study extensively in plants by MALDI-MSI ([Horn et al., 2012](#_ENREF_5), [Sturtevant et al., 2017](#_ENREF_10)). The high abundance of PL-related signals in soybean nodule system is in accordance with previous estimates suggesting that plant membrane biogenesis needs to be upregulated several fold in order to provide for infection thread development, symbiosme membrane formation, and other membranes involved in nodule development and function ([Roth and Stacey, 1989](#_ENREF_8)). In positive ion mode we were able to map [M+H]^+^ and [M+K]^+^ pseudomolecular ion species of phosphatidic acids (PA), phosphatidylethanolamines (PE), and phosphatidylcholines (PC). All PLs detected in positive ion mode showed co-localization with the infection region, but with different distribution patterns. Using negative ion mode, we additionally determined phophatydilinositols (PI), phosphatydilglycerols (PG), and phosphatydilserines (PS) co-localized either with cortex or in specific compartments of infection zone. Some PLs were detected in both positive and negative ion mode, like PA (36:2), PE (34:2), and PE (34:1). In each case, their localization was consistent between polarities, showing the robustness and verity of the visualization method. Interestingly, we observed that even inside a single class of PLs large distributional differences were measurable. A striking example of this is where PAs were co-localized with the cortex (PA 32:0), uniformly distributed through the entire section (PA 36:4, PA 40:2), asymmetrically distributed within the infection zone (PA 36:4, PA 36:3), or showed centralized distribution pattern (PA 34:1, PA 36:2, PA 38:3, PA 36:2). Such differences in compartmentalization of PL suggests that metabolic pathways for these metabolites occurred heterogeneously within the soybean nodule.


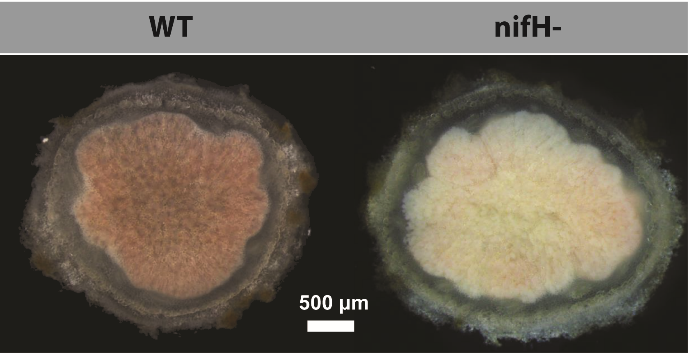


**Figure S4.** Optical images of cross-sections of WT and *nif*H- soybean nodules showing the differences in color of the infection zone between strains. The reddish color is a result of the presence of legheamoglobin’s chromophore (*heme* B).


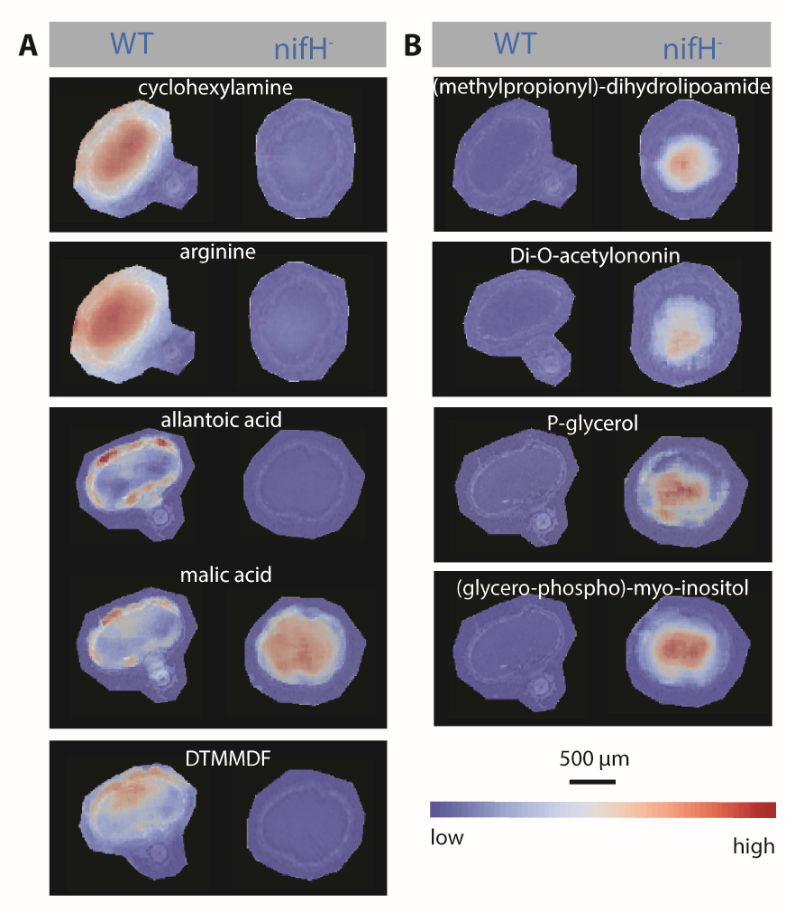


**Figure S5.** Distributions and abundances of metabolites that are measurably different between WT and nifH^-^ mutant soybean nodules. The rest of metabolites annotated in Figure S2 show similar distribution patterns and abundances between two strains. (**a**) Metabolites imaged almost exclusively in WT strain. Although not differently distributed between two strains, malic acid is depicted together with allantoic acid to provide general insight into differences in C source input and N source output of symbiotic system, respectively. These results suggest that host doesn’t sanction ineffective nodules by limiting their energy supply, given that malic acid was not found as discriminant molecule between WT and nifH- mutant. Therefore, the hypothesis that limitation in oxygen is main host-sanction mechanism ([Kiers et al., 2003](#_ENREF_6)) is indirectly supported by our study. (**b**) Metabolites imaged almost exclusively in nifH^-^ mutant. Each WT and nifH^-^ pair is analyzed in the same experiment with the same image adjustment processing.

**Table S1.** Peak assignments in positive-ion mode profiling MALDI-FTICR mass spectra of soybean root nodules. Metabolites are detected as [M+H]^+^ species unless otherwise is specified. Levels of confidence: Level 1: identified compounds based on ultra-high mass accuracy (<1 ppm) and at least one more independent orthogonal data (tandem MS or collision cross section (ccs)); Level 2a: Putatively annotated compounds based upon ultra-high mass accuracy (<1 ppm) and soybean/legume nodule literature coverage; Level 2b: Putatively annotated compounds according solely to ultra-high mass accuracy (<1 ppm) as was originally defined by the Metabolomics Standard Initiative.([Sumner et al., 2007](#_ENREF_11))

| **Metabolite** | **Measured** | **Theoretical** | **Error** | **MSI level** | **Localization** |
| --- | --- | --- | --- | --- | --- |
|  | **m/z** | **m/z** | **(ppm)** |  |  |
| Cyclohexylamine ^a^ | 100.1121 | 100.1121 | 0.00 | 1 | Infection r |
| Choline ^a,b^ | 104.1070 | 104.1070 | 0.00 | 1 | Infection r |
| Coniceine | 126.1278 | 126.1277 | 0.79 | 2b | Infection r |
| Leucine ^b^ | 132.1020 | 132.1019 | 0.76 | 2a | Infection r |
| Asparagine ^c^ | 133.0608 | 133.0608 | 0.00 | 2a | Cortex |
| Adenine ^a^ | 136.0619 | 136.0620 | 0.73 | 1 | Infection r |
| Methylnicotineamide | 137.0710 | 137.0709 | 0.73 | 2b | Infection r |
| Guanidinobutanoic acid | 146.0925 | 146.0924 | 0.68 | 2b | Infection r |
| Spermidine ^a^ | 146.1653 | 146.1652 | 0.68 | 1 | Infection r |
| Guanine ^a^ | 152.0568 | 152.0567 | 0.66 | 1 | Infection r |
| Histidine ^b^ | 156.0768 | 156.0768 | 0.00 | 2a | Infection r |
| Homostachydrine/Lentiginosine | 158.1176 | 158.1176 | 0.00 | 2b | Infection r |
| Guanidinovaleric acid | 160.1081 | 160.1081 | 0.00 | 2b | Infection r |
| Homospermidine ^d^ | 160.1809 | 160.1808 | 0.62 | 2a | Infection r |
| Phosphonoacetaldehyde+K | 162.9558 | 162.9557 | 0.61 | 2b | Infection r |
| Aminobutyl cadaverin (aminobutyl-cad) | 174.1966 | 174.1965 | 0.57 | 2b | Infection r |
| Arginine ^a,b,c^ | 175.1190 | 175.1190 | 0.00 | 1 | Infection r |
| Calystegin | 176.0918 | 176.0917 | 0.57 | 2b | Infection r |
| Citruline ^a^ | 176.1031 | 176.1030 | 0.57 | 1 | Infection r |
| Phosphocholine ^a^ | 184.0734 | 184.0733 | 0.54 | 1 | Infection r |
| Diaminononanoate+K | 189.1599 | 189.1598 | 0.53 | 2b | Infection r |
| Aminooctanoic acid+K | 198.0891 | 198.0891 | 0.00 | 2b | Infection r |
| Spermine | 203.2231 | 203.2230 | 0.49 | 2b | Infection r |
| Acetylcarnitine | 204.1231 | 204.1230 | 0.49 | 2b | Infection r |
| Kinetin | 216.0881 | 216.0880 | 0.46 | 2b | Infection r |
| Solamine | 216.2435 | 216.2434 | 0.46 | 2b | Infection r |
| Acetyldihydrolipoamide +H-H2O | 232.0830 | 232.0830 | 0.00 | 2b | Infection r |
| Securinine+Na | 240.0995 | 240.0995 | 0.00 | 2b | Infection r |
| Adenosine ^a^ | 268.1042 | 268.1040 | 0.75 | 1 | Infection r |
| Deoxy(methylthio)adenosine | 298.0970 | 298.0968 | 0.67 | 2b | Infection r |
| Graveoline/avenalumin II +Na | 302.0787 | 302.0788 | 0.33 | 2b | Infection r |
| Harzianopyridone+K | 320.0893 | 320.0895 | 0.62 | 2b | Infection r |
| Guanosine+K | 322.0547 | 322.0548 | 0.31 | 2b | Infection r |
| coumarin glucosides | 363.0689 | 363.0687 | 0.55 | 2b | Cortex |
| Disaccharide +K ^a,b^ | 381.0793 | 381.0794 | 0.26 | 1 | Cortex Inner |
| S-adenosyl methionine ^a,c^ | 399.1444 | 399.1445 | 0.25 | 1 | Infection r |
| Sucrose-phosphate | 423.0900 | 423.0898 | 0.47 | 2b | Cortex Outer |
| ADP ^a^ | 428.0366 | 428.0367 | 0.23 | 1 | Infection r |
| Trihydroxyflavon glucoside + Na ^a^ | 471.0899 | 471.0898 | 0.21 | 1 | Cortex Inner |
| Adenylosuccinate+K | 502.0376 | 502.0372 | 0.80 | 2b | Cortex Inner |
| Tetrahydroxymethoxyflavone glucoside + K | 517.0954 | 517.0953 | 0.19 | 2b | Cortex Inner |
| CDP-choline +K | 527.0709 | 527.0705 | 0.76 | 2b | Cortex Inner |
| Apigenin(acetyl-methylglucuronide)+K | 541.0743 | 541.0743 | 0.00 | 2b | Cortex |
| cADP-ribose ^a^ | 542.0683 | 542.0684 | 0.18 | 1 | Infection r |
| ATP-propionic acid ^c^ | 580.0241 | 580.0242 | 0.17 | 2a | Infection r |
| Heme *B* ^a,b,c^ | 616.1766 | 616.1768 | 0.32 | 1 | Infection r |
| PA (36:4)+K | 735.4362 | 735.4362 | 0.00 | 2b | Infection r |
| PA (36:3)+K | 737.4520 | 737.4518 | 0.27 | 2b | Infection r |
| PA (36:2)+K | 739.4675 | 739.4675 | 0.00 | 2b | Infection r |
| PE (34:2)+K | 754.4783 | 754.4784 | 0.13 | 2b | Infection r |
| PE (34:1)+K | 756.4942 | 756.4940 | 0.26 | 2b | Infection r |
| PC(34:3) | 756.5538 | 756.5538 | 0.00 | 2b | Infection r |
| PC (34:2) /MMPE(36:2) | 758.5695 | 758.5694 | 0.13 | 2b | Infection r |
| PC (34:1) | 760.5853 | 760.5851 | 0.26 | 2b | Infection r |
| PA (38:4)+K | 763.4676 | 763.4675 | 0.13 | 2b | Infection r |
| PA (38:3)+K | 765.4831 | 765.4831 | 0.00 | 2b | Infection r |
| PC (32:1)+K/MMPE(34:1)+K | 770.5097 | 770.5097 | 0.00 | 2b | Infection r |
| PE (38:1) | 774.6007 | 774.6007 | 0.00 | 2b | Infection r |
| PE (36:2)+K | 782.5099 | 782.5097 | 0.26 | 2b | Infection r |
| PC(36:3) | 784.5852 | 784.5851 | 0.13 | 2b | Infection r |
| PC(36:2) | 786.6008 | 786.6007 | 0.13 | 2b | Infection r |
| PC (34:2)+K/MMPE(36:2)+K | 796.5254 | 796.5253 | 0.13 | 2b | Infection r |
| PC(34:1)+K | 798.5411 | 798.5410 | 0.13 | 2b | Infection r |
| PE(40:2) | 800.6162 | 800.6164 | 0.25 | 2b | Infection r |
| PE(38:2)+K | 810.5410 | 810.5409 | 0.12 | 2b | Infection r |
| PE (38:1)+K | 812.5564 | 812.5566 | 0.25 | 2b | Infection r |
| PC (38:2) | 814.6317 | 814.6320 | 0.37 | 2b | Infection r |
| PC (36:5) +K | 818.5093 | 818.5097 | 0.49 | 2b | Infection r |
| PC (36:4)+K | 820.5253 | 820.5253 | 0.00 | 2b | Infection r |
| PC (36:3) + K | 822.5409 | 822.5410 | 0.12 | 2b | Infection r |
| PC (36:2)+K | 824.5564 | 824.5566 | 0.24 | 2b | Infection r |
| PE(40:2)+K | 838.5719 | 838.5721 | 0.24 | 2b | Infection r |
| PC(38:2)+K | 852.5875 | 852.5879 | 0.47 | 2b | Infection r |
| Soyasaponin II +K ^a^ | 951.4717 | 951.4714 | 0.32 | 1 | Cortex Outer |
| Dehydrosoyasaponin I +K ^a^ | 979.4666 | 979.4663 | 0.31 | 1 | Cortex Outer |
| Soyasaponin I +K ^a^ | 981.4822 | 981.4820 | 0.20 | 1 | Cortex Outer |

^a^ Chemical species assigned based on in-house LAESI MSMS and/or ion mobility results.([Stopka et al., 2017](#_ENREF_9))

^b^ Chemical species assigned based on ([Ye et al., 2013](#_ENREF_13))

^c^ Chemical species assigned based on ([Gemperline et al., 2015](#_ENREF_4))

^d^ Chemical species assigned based on ([Vauclare et al., 2013](#_ENREF_12))

^e^ Chemical species assigned based on ([Brechenmacher et al., 2010](#_ENREF_2)).

**Table S2.** Peak assignments in negative-ion mode profiling MALDI FTICR mass spectra of soybean root nodules. Metabolites are detected as [M-H]^-^ species. Levels of confidence: Level 1: identified compounds based on ultra-high mass accuracy (<1ppm) and at least one more independent orthogonal data (tandem MS or collision cross section (ccs)); Level 2a: Putatively annotated compounds based upon ultra-high mass accuracy (<1ppm) and soybean/legume nodule literature coverage; Level 2b: Putatively annotated compounds according solely to ultra-high mass accuracy (<1ppm) as was originally defined by the Metabolomics Standard Initiative.([Sumner et al., 2007](#_ENREF_11))

| **Metabolite** | **Measured** | **Theoretical** | **Error** | **MSI level** | **Localization** |
| --- | --- | --- | --- | --- | --- |
|  | **m/z** | **m/z** | **(ppm)** |  |  |
| Sulfuric acid | 96.9601 | 96.9601 | 0.00 | 2b | Cortex |
| Succinic acid ^b^ | 117.0193 | 117.0193 | 0.00 | 2a | Cortex |
| Asparagine ^c^ | 131.0462 | 131.0462 | 0.00 | 2a | Cortex |
| Malic acid ^a,b^ | 133.0143 | 133.0142 | 0.75 | 1 | Infection r |
| Glutamate ^a c d e^ | 146.0458 | 146.0459 | 0.68 | 1 | Infection r |
| Pentose ^a,b^ | 149.0456 | 149.0455 | 0.67 | 1 | Cortex |
| Vanillic acid ^a^ | 167.0350 | 167.0350 | 0.00 | 1 | Cortex |
| Ascorbic acid ^b^ | 175.0248 | 175.0248 | 0.00 | 2a | Infection r |
| Allantoic acid ^e^ | 175.0473 | 175.0473 | 0.00 | 2a | Cortex |
| Methoxycinnamic acid | 177.0558 | 177.0557 | 0.56 | 2b | Cortex |
| Hydroxyohenylpyruvic acid | 179.0351 | 179.0350 | 0.56 | 2b | Cortex |
| Homovanillic acid | 181.0507 | 181.0506 | 0.55 | 2b | Cortex |
| Acetyl-Glutamic acid | 188.0565 | 188.0564 | 0.53 | 2b | Cortex |
| Citric acid | 191.0197 | 191.0197 | 0.00 | 2b | Cortex |
| Ferulic acid ^e^ | 193.0507 | 193.0506 | 0.52 | 2a | Cortex |
| Gluconic acid ^a^ | 195.0511 | 195.0510 | 0.51 | 1 | Cortex |
| Me-citrate/homoisocitrate | 205.0354 | 205.0354 | 0.00 | 2b | Infection r |
| Glucarate | 209.0303 | 209.0303 | 0.00 | 2b | Cortex |
| Trimethoxycoumarines | 235.0613 | 235.0612 | 0.43 | 2b | Cortex |
| Inositol cyclic phosphate | 241.0119 | 241.0119 | 0.00 | 2b | Cortex |
| N-Feruloylglycine | 250.0721 | 250.0721 | 0.00 | 2b | Cortex |
| Dihydroxyflavone ^a^ | 253.0507 | 253.0506 | 0.40 | 1 | Cortex |
| Hex-phosphate ^a,b^ | 259.0224 | 259.0224 | 0.00 | 1 | Infection r |
| Trihydroxyflavone ^a^ | 269.0457 | 269.0455 | 0.74 | 1 | Cortex |
| Dihydroxytetramethoxy methylendioxy flavone (DTMMDF) ^a^ | 417.0828 | 417.0827 | 0.24 | 1 | Infection r |
| flavon malonyl glucoside ^a^ | 485.1089 | 485.1089 | 0.00 | 1 | Cortex Outer |
| UDP-hexose ^a,b^ | 565.0477 | 565.0477 | 0.00 | 1 | Infection r |
| Flavonoid diglycoside | 595.1305 | 595.1305 | 0.00 | 2b | Cortex Inner |
| UDP-NAcGlcN  ^a^ | 606.0742 | 606.0743 | 0.16 | 1 | Infection r |
| PS (24:0) | 622.3726 | 622.3725 | 0.16 | 2b | Cortex Inner |
| PA (32:0) | 647.4656 | 647.4657 | 0.15 | 2b | Cortex Inner |
| PG (28:1) | 663.4242 | 663.4243 | 0.15 | 2b | Infection r |
| PA (34:3) | 669.4500 | 669.4501 | 0.15 | 2b | Cortex Inner |
| PA (34:2) | 671.4656 | 671.4657 | 0.15 | 2b | Cortex Inner |
| PA (34:1) | 673.4813 | 673.4814 | 0.15 | 2b | Infection r |
| PS(28:1) | 676.4195 | 676.4195 | 0.00 | 2b | Infection r |
| PA (36:5) | 693.4498 | 693.4501 | 0.43 | 2b | Cortex Inner |
| PA (36:4)^a^ | 695.4657 | 695.4657 | 0.00 | 1 | Cortex Inner |
| PA(36:2) | 699.4969 | 699.4970 | 0.14 | 2b | Infection r |
| PE (34:2)^a^ | 714.5078 | 714.5080 | 0.28 | 1 | Infection r |
| PE (34:1)^a^ | 716.5234 | 716.5236 | 0.28 | 1 | Infection r |
| PG(32:0) | 721.5024 | 721.5025 | 0.14 | 2b | Infection r |
| PA(38:3) | 725.5126 | 725.5127 | 0.14 | 2b | Infection r |
| PA(38:2) | 727.5282 | 727.5283 | 0.14 | 2b | Infection r |
| PE(36:5) | 736.4922 | 736.4923 | 0.14 | 2b | Infection r |
| PE(36:4) | 738.5079 | 738.5079 | 0.00 | 2b | Infection r |
| PE(36:3) | 740.5235 | 740.5236 | 0.14 | 2b | Infection r |
| PG(34:2) | 745.5024 | 745.5025 | 0.13 | 2b | Infection r |
| PG (34:1)^a^ | 747.5181 | 747.5182 | 0.13 | 1 | Infection r |
| PA(40:3) | 753.5439 | 753.5440 | 0.13 | 2b | Infection r |
| PA(40:2) | 755.5595 | 755.5596 | 0.13 | 2b | Infection r |
| PS(34:3) | 756.4819 | 756.4821 | 0.26 | 2b | Infection r |
| PS(34:2) | 758.4975 | 758.4978 | 0.40 | 2b | Infection r |
| PS (34:1) | 760.5131 | 760.5134 | 0.39 | 2b | Infection r |
| PI(30:4) | 773.4241 | 773.4247 | 0.78 | 2b | Cortex Inner |
| PG (36:2)^a^ | 773.5336 | 773.5338 | 0.26 | 1 | Infection r |
| PI(32:0) | 809.5185 | 809.5186 | 0.12 | 2b | Cortex Inner |
| PI(34:3) | 831.5027 | 831.5029 | 0.24 | 2b | Cortex Inner |
| PI(34:2) | 833.5185 | 833.5186 | 0.12 | 2b | Cortex Inner |
| PS(40:2) | 842.5914 | 842.5917 | 0.36 | 2b | Infection r |
| Soyasaponin II ^a^ | 911.5015 | 911.5010 | 0.55 | 1 | Cortex Outer |
| Dehydrosoyasaponin I ^a^ | 939.4960 | 939.4958 | 0.21 | 1 | Cortex Outer |
| Soyasaponin I ^a^ | 941.5124 | 941.5115 | 0.96 | 1 | Cortex Outer |

^a^ Chemical species assigned based on in-house LAESI MSMS and/or ion mobility results.([Stopka et al., 2017](#_ENREF_9))

^b^ Chemical species assigned based on ([Ye et al., 2013](#_ENREF_13))

^c^ Chemical species assigned based on ([Gemperline et al., 2015](#_ENREF_4))

^d^ Chemical species assigned based on ([Vauclare et al., 2013](#_ENREF_12))

^e^ Chemical species assigned based on ([Brechenmacher et al., 2010](#_ENREF_2))

**Table S3.** MALDI-FTICR-MSI metabolic coverage of some pathways in soybean root nodule based on SoyKB database.

| Pathway | Kegg | Compound | % coverage |
| --- | --- | --- | --- |
| Purine metabolism | cpd:C00008 | ADP | 13.1 |
|  | cpd:C00212 | Adenosine |  |
|  | cpd:C00147 | Adenine |  |
|  | cpd:C03794 | Adenylosuccinate |  |
|  | cpd:C00499 | Allantoic acid |  |
|  | cpd:C00059 | Sulfuric acid |  |
|  | cpd:C00242 | Guanine |  |
|  | cpd:C00387 | Guanosine |  |
| Glyoxylate and dicarboxylate metabolism | cpd:C00158 | Citric acid | 17.7 |
|  | cpd:C00149 | Malic acid |  |
|  | cpd:C00042 | Succinic acid |  |
| Zeatin biosynthesis | cpd:C00008 | ADP | 15.8 |
|  | cpd:C00147 | Adenine |  |
|  | cpd:C00029 | Uridine diphosphate glucose |  |
| Citrate cycle (TCA cycle) | cpd:C00149 | Malic acid | 20 |
|  | cpd:C00042 | Succinic acid |  |
|  | cpd:C00158 | Citric acid |  |
|  | cpd:C16255 | S-Acetyldihydrolipoamide |  |
| Arginine and proline metabolism | cpd:C00327 | Citrulline | 10.5 |
|  | cpd:C00062 | L-Arginine |  |
|  | cpd:C00025 | Glutamate |  |
|  | cpd:C00624 | N-Acetyl-L-glutamic acid |  |
|  | cpd:C00315 | Spermidine |  |
|  | cpd:C00750 | Spermine |  |
| beta-Alanine metabolism | cpd:C00315 | Spermidine | 16.6 |
|  | cpd:C00750 | Spermine |  |
| Glutathione metabolism | cpd:C00025 | Glutamate | 12.3 |
|  | cpd:C00315 | Spermidine |  |
|  | cpd:C00072 | Ascorbic acid |  |
|  | cpd:C00750 | Spermine |  |
| Aminoacyl-tRNA biosynthesis | cpd:C00025 | Glutamate | 7.5 |
|  | cpd:C00062 | L-Arginine |  |
|  | cpd:C00152 | L-Asparagine |  |
|  | cpd:C00135 | L-Histidine |  |
|  | cpd:C00123 | L-Leucine |  |
| Aminoacyl-tRNA biosynthesis | cpd:C00818 | D-Glucarate | 20 |
|  | cpd:C00072 | Ascorbic acid |  |
|  | cpd:C0029 | Uridine diphosphate glucose |  |
| Histidine metabolism | cpd:C05575 | Hercynine | 18.8 |
|  | cpd:C00025 | Glutamate |  |
|  | cpd:C00025 | Hercynine |  |
| Glycerophospholipid metabolism | cpd:C00307 | CDP-choline | 12 |
|  | cpd:C00588 | Phosphocholine |  |
|  | cpd:C00114 | Choline |  |
| Alanine, aspartate and glutamate metabolism | cpd:C00042 | Succinic acid | 22.7 |
|  | cpd:C03794 | Adenylosuccinate |  |
|  | cpd:C00025 | Glutamate |  |
|  | cpd:C00158 | Citric acid |  |
|  | cpd:C00152 | L-Asparagine |  |
| Tyrosine metabolism | cpd:C00042 | Succinic acid | 16.7 |
|  | cpd:C05582 | Homovanillic acid |  |
|  | cpd:C01179 | 4-Hydroxyphenylpyruvic acid |  |
| Nitrogen metabolism | cpd:C00025 | Glutamate | 6.7 |
| Starch and sucrose metabolism | cpd:C16688 | Sucrose-6-phosphate | 10 |
|  | cpd:C00089 | Sucrose |  |
|  | cpd:C00029 | Uridine diphosphate glucose |  |

**Table S4.** The average Pearson’s correlation coefficients of SAM, ADP, and *heme* B. Pearson’s correlation coefficients were calculated using the SCILS software.

|  | **SAM** | **ADP** | ***heme* B** |
| --- | --- | --- | --- |
| **SAM** | - | 0.80 ± 0.09 | 0.62 ± 0.09 |
| **ADP** | 0.80 ± 0.09 | - | 0.62 ± 0.12 |
| ***heme* B** | 0.62 ± 0.09 | 0.62 ± 0.12 | - |
| **Nodule shape** | 0.58 ± 0.12 | 0.59 ± 0.09 | 0.66 ± 0.13 |
|  |  |  |  |

**References**

Anderton, C. R., Chu, R. K., Tolic, N., Creissen, A. and Pasa-Tolic, L. (2016). Utilizing a Robotic Sprayer for High Lateral and Mass Resolution MALDI FT-ICR MSI of Microbial Cultures. Journal of the American Society for Mass Spectrometry **27**: 556-559.

Brechenmacher, L., Lei, Z. T., Libault, M., Findley, S., Sugawara, M., Sadowsky, M. J., Sumner, L. W. and Stacey, G. (2010). Soybean Metabolites Regulated in Root Hairs in Response to the Symbiotic Bacterium Bradyrhizobium japonicum. Plant Physiology **153**: 1808-1822.

Cole, M. A. and Elkan, G. H. (1973). Transmissible Resistance to Penicillin-G, Neomycin, and Chloramphenicol in Rhizobium-Japonicum. Antimicrobial Agents and Chemotherapy **4**: 248-253.

Gemperline, E., Jayaraman, D., Maeda, J., Ane, J. M. and Li, L. (2015). Multifaceted investigation of metabolites during nitrogen fixation in Medicago via high resolution MALDI-MS imaging and ESI-MS. J Am Soc Mass Spectrom **26**: 149-158.

Horn, P. J., Korte, A. R., Neogi, P. B., Love, E., Fuchs, J., Strupat, K., Borisjuk, L., Shulaev, V., Lee, Y. J. and Chapman, K. D. (2012). Spatial Mapping of Lipids at Cellular Resolution in Embryos of Cotton. Plant Cell **24**: 622-636.

Kiers, E. T., Rousseau, R. A., West, S. A. and Denison, R. F. (2003). Host sanctions and the legume-rhizobium mutualism. Nature **425**: 78-81.

Palmer, A., Phapale, P., Chernyavsky, I., Lavigne, R., Fay, D., Tarasov, A., Kovalev, V., Fuchser, J., Nikolenko, S., Pineau, C., Becker, M. and Alexandrov, T. (2017). FDR-controlled metabolite annotation for high-resolution imaging mass spectrometry. Nature Methods **14**: 57-60.

Roth, L. E. and Stacey, G. (1989). Bacterium Release into Host-Cells of Nitrogen-Fixing Soybean Nodules - the Symbiosome Membrane Comes from 3 Sources. European Journal of Cell Biology **49**: 13-23.

Stopka, S. A., Agtuca, B. J., Koppenaal, D. W., Pasa-Tolic, L., Stacey, G., Vertes, A. and Anderton, C. R. (2017). Laser Ablation Electrospray Ionization Mass Spectrometry with Ion Mobility Separation Reveals Metabolites in the Symbiotic Interactions of Soybean Roots and Rhizobia. Plant J.

Sturtevant, D., Duenas, M. E., Lee, Y. J. and Chapman, K. D. (2017). Three-dimensional visualization of membrane phospholipid distributions in Arabidopsis thaliana seeds: A spatial perspective of molecular heterogeneity. Biochimica Et Biophysica Acta-Molecular and Cell Biology of Lipids **1862**: 268-281.

Sumner, L. W., Amberg, A., Barrett, D., Beale, M. H., Beger, R., Daykin, C. A., Fan, T. W. M., Fiehn, O., Goodacre, R., Griffin, J. L., Hankemeier, T., Hardy, N., Harnly, J., Higashi, R., Kopka, J., Lane, A. N., Lindon, J. C., Marriott, P., Nicholls, A. W., Reily, M. D., Thaden, J. J. and Viant, M. R. (2007). Proposed minimum reporting standards for chemical analysis. Metabolomics **3**: 211-221.

Vauclare, P., Bligny, R., Gout, E. and Widmer, F. (2013). An overview of the metabolic differences between Bradyrhizobium japonicum 110 bacteria and differentiated bacteroids from soybean (Glycine max) root nodules: an in vitro 13C-and 31P-nuclear magnetic resonance spectroscopy study. Fems Microbiology Letters **343**: 49-56.

Ye, H., Gemperline, E., Venkateshwaran, M., Chen, R., Delaux, P. M., Howes-Podoll, M., Ane, J. M. and Li, L. (2013). MALDI mass spectrometry-assisted molecular imaging of metabolites during nitrogen fixation in the Medicago truncatula-Sinorhizobium meliloti symbiosis. Plant J **75**: 130-145.

Zabrouskov, V., Al-Saad, K. A., Siems, W. F., Hill, H. H., Jr. and Knowles, N. R. (2001). Analysis of plant phosphatidylcholines by matrix-assisted laser desorption/ionization time-of-flight mass spectrometry. Rapid Commun Mass Spectrom **15**: 935-940.
